# Oxidative stress and impaired cardiovagal baroreflex sensitivity in young adults with post‐traumatic stress disorder

**DOI:** 10.1113/EP093755

**Published:** 2026-04-25

**Authors:** Jennifer B. Weggen, Jacob W. Richardson, Emily A. Buck, Brad T. Bakken, Brandon J. Fitzpatrick, Raven G. Campbell, Ryan S. Garten

**Affiliations:** ^1^ Department of Kinesiology and Health Sciences Virginia Commonwealth University Richmond Virginia USA

**Keywords:** autonomic dysfunction, baroreflex sensitivity, cardiovascular disease risk, oxidative stress, post‐traumatic stress disorder

## Abstract

Post‐traumatic stress disorder (PTSD) is associated with elevated cardiovascular disease (CVD) risk, yet the underlying physiological characterization in young adults remain unclear. This study examines whether autonomic and/or vascular dysfunction predominates as an early precursor to CVD development in young adults with PTSD and explores the potential contribution of oxidative stress (OXS). Forty‐four young adults (22 PTSD, 22 controls; 19F/3M per group) free from cardiometabolic disease were evaluated for OXS, autonomic regulation and vascular function. OXS was quantified via electron paramagnetic resonance analysis of whole blood superoxide concentration. Autonomic function was assessed using an isometric handgrip exercise (exercise pressor reflex) and a Valsalva manoeuvre to determine cardiovagal baroreflex sensitivity (cBRS). Local vascular control was evaluated through rapid onset vasodilation (ROV) in response to a brief forearm contraction. Compared with controls, the PTSD group exhibited significantly higher resting systolic (*P* < 0.01) and mean arterial pressure (*P* = 0.04) and elevated superoxide levels (*P* < 0.01). cBRS was markedly reduced in PTSD during Phase IV of the Valsalva manoeuvre (*P* < 0.01), indicating impaired autonomic regulation. In contrast, vascular conductance and blood flow responses to the ROV and exercise pressor tests were preserved. These findings suggest that OXS and baroreflex dysfunction, rather than local vascular impairment, represent early detectable maladaptations potentially contributing to heightened CVD risk in young adults with PTSD. Early identification and mitigation of oxidative and autonomic imbalances may help prevent premature cardiovascular decline in this population.

## Abstract (highlights)

- **What is the central question of this study?**

Are oxidative stress, autonomic regulation and vascular function altered in young adults with PTSD?

- **What is the main finding and its importance?**

Young adults with PTSD exhibited elevated oxidative stress and reduced cardiovagal baroreflex sensitivity compared with controls. In contrast, vascular conductance and exercise pressor responses during isometric handgrip were preserved. These findings demonstrate the coexistence of oxidative stress and impaired cardiovagal baroreflex function in young adults with PTSD despite maintained peripheral vascular responses, providing insight into physiological alterations associated with PTSD that may be relevant to early cardiovascular risk.

## 1 INTRODUCTION

Post‐traumatic stress disorder (PTSD) is a complex mental health condition with profound physiological implications, particularly affecting cardiovascular health. While extensive research has been conducted on its psychological effects, growing evidence links PTSD to significant disruptions in autonomic regulation, often characterized by an overactive sympathetic nervous system and diminished parasympathetic function (Fu, 2022). These autonomic disturbances are often observed as altering resting heart rate (HR) and blood pressure (Buckley & Kaloupek, 2001) as well as poor blood pressure control (Edmondson et al., 2018; Fonkoue, Norrholm, et al., 2018) leading to inappropriate elevations in response to normal stimuli, all of which raise the likelihood of CVD, such as hypertension and heart disease. The elevated CVD risk is further amplified by increased oxidative stress (OXS) in individuals with PTSD (Ceprnja et al., 2011; Weggen et al., 2021) with chronic activation of the stress response leading to excessive production of reactive oxygen species (ROS) (Liu et al., 2025). This excess production of ROS damages cellular structures, promotes inflammation and impairs vascular function, thereby compounding the physiological burden imposed by PTSD and contributing to further deterioration of cardiovascular health (Sidik et al., 2025). It should be noted that the evidence of autonomic imbalance, vascular dysfunction and OXS among individuals with PTSD is primarily observed in studies focused on middle‐aged and older adults, which can be confounded by age‐related cardiovascular changes and prolonged consequences of PTSD‐associated lifestyle behaviours (e.g., smoking, drug abuse, sedentary behaviour and obesity) and medication use.

Prior work by our laboratory (Weggen et al., 2021, 2024) deliberately focused on young adults (18–35 years) to limit common confounding variables, enabling a more direct assessment of the impact of PTSD symptomatology on cardiovascular health. This work has identified the presence of cardiovascular dysfunction in young adults with PTSD, but was not designed to determine if this dysfunction was driven by mechanisms derived from the autonomic nervous system or local vascular control. Indeed, our group identified lower flow‐mediated dilation (Weggen et al., 2021), a factor that can be altered by loss of local vasodilators such as nitric oxide, as well as elevated sympathetic nervous system (SNS) activity at rest (Gundersen et al., 2019; Wray et al., 2017). Further, our group identified lower vascular conductance during handgrip exercise that was linked to elevated exercise‐induced blood pressure responses (Weggen et al., 2024). This latter finding may be driven by reductions in local vasodilation and/or elevated, inappropriate blood pressure responses secondary to sensitization of skeletal muscle afferents, baroreflex dysfunction and sensitization of adrenergic receptors on the vascular smooth muscle, factors reported previously in middle‐aged and older individuals with PTSD (D'Souza et al., 2024; Fonkoue et al., 2020; Park et al., 2017). Further, although not directly measured, both studies from our laboratory implicated OXS in the observed dysfunction; specifically, Weggen et al. (2021) observed partial improvement in upper limb flow‐mediated dilation, while Weggen et al. (2024) noted similar improvements in exercise‐induced vascular conductance following acute antioxidant supplementation.

Together, these findings underscore a critical need to delineate which specific cardiovascular maladaptations are present in young individuals with PTSD, thereby identifying potential early physiological markers of elevated cardiovascular disease (CVD) risk in this population. Thus, the main objective of this study was to further examine how PTSD influences cardiovascular function in young adults, particularly examining the distinct roles of vascular and autonomic function, as well as directly assessing OXS in this population. We adopted a multifaceted approach assessing whole blood superoxide, cardiovagal baroreflex sensitivity (cBRS), exercise pressor responses, and rapid onset vasodilation (ROV) along with minimizing confounding factors such as negative lifestyle behaviours and ageing by selecting a young cohort of adults with PTSD and comparing them to an age‐ and sex‐matched control group to assess these gaps. This approach enabled us to isolate the independent physiological effects of PTSD. We hypothesized that young adults with PTSD would exhibit elevated OXS accompanied by impairments in autonomic and vascular function compared with age‐ and sex‐matched controls. Specifically, we hypothesized that individuals with PTSD would demonstrate higher whole blood superoxide levels, autonomic dysregulation (exacerbated exercise pressor responses and reduced cBRS), and attenuated peripheral vascular responses.

## 2 METHODS

### 2.1 Ethical approval

The study followed the ethical standards of the *Declaration of Helsinki*, except for registration in a database. Informed consent from all participants was obtained in writing, and all procedures were approved (HM20020955) by the Institutional Review Board of Virginia Commonwealth University.

### 2.2 Participants

Forty‐four young adults with (*n* = 22; 19F/3M) and without (*n* = 22; 19F/3M) PTSD were recruited through online campus newsletters, word of mouth and posted flyers. All participants were screened using a self‐reported medical history questionnaire completed prior to the preliminary visit. Based on these responses, participants were classified as apparently healthy and free from cardiovascular, pulmonary or metabolic diseases. Eligibility for the PTSD group was evaluated using the Posttraumatic Stress Checklist for the DSM‐5 (PCL‐5; Diagnostics and Statistical Manual of Mental Disorders, 5th Edition) or self‐reported prior diagnosis. This validated self‐report incorporates 20 five‐point (0–4) Likert scales, with total scores between 0 and 80. For inclusion in the PTSD group, participants must score ≥33 on the PCL‐5, a cutoff within the validated 31–33 range that demonstrates strong diagnostic accuracy when compared to the gold standard Clinician‐Administered PTSD Scale‐5 (CAPS‐5) (Blevins et al., 2015; Bovin et al., 2016). Exclusion criteria were assessed through an extensive medical history questionnaire completed during the preliminary screening visit. Participants were asked to disclose the use of prescribed medication (Table 1) but were advised to continue using them as prescribed during the study. No participant disclosed any smoking, tobacco use or illicit drug use.

**TABLE 1:** Subject characteristics.

	CTRL (*n* = 22)	PTSD (*n* = 22)	*P*
Sex (female/male)	19/3	19/3	
Age (years)	24 ± 5	25 ± 6	0.48
Height (cm)	164 ± 9	166 ± 8	0.58
Weight (kg)	66 ± 17	70 ± 18	0.51
Body fat (%)	28 ± 10	27 ± 6	0.84
BMI (kg/m^2^)	24 ± 5	25 ± 6	0.51
MVC (kg)	28 ± 9	29 ± 8	0.75
Systolic blood pressure	114 ± 8	122 ± 11*	<0.01
Diastolic blood pressure	69 ± 7	73 ± 8	0.13
Mean arterial pressure	84 ± 8	89 ± 8*	0.04
Heart rate	71 ± 12	74 ± 12	0.44
Mental health assessments			
Beck anxiety	10 ± 8	29 ± 10*	<0.01
Beck depression	7 ± 5	22 ± 10*	<0.01
Trait anxiety	41 ± 11	55 ± 10*	<0.01
PCL‐5 Score	9 ± 8	43 ± 13*	<0.01
Medications, *n* (%)			
Selective serotonin reuptake inhibitor	0 (0)	3 (23)	
Anti‐convulsant	0 (0)	1 (8)	
NA and dopamine reuptake inhibitor	0 (0)	1 (8)	
CNS stimulant	0 (0)	1 (8)	

Values are expressed as means ± SD or *n* (%). Statistical analysis was conducted using an independent samples *t*‐test (CTRL vs. PTSD).

*Significantly different that CTRL group (*P* < 0.05). BMI, body mass index; CTRL, control; MVC, maximal voluntary contraction; NA, noradrenaline; PTSD, post‐traumatic stress disorder.

### 2.3 Familiarization visit

All qualified participants took part in a familiarization visit. The main goal of this visit was to reduce participants’ stress and state anxiety by increasing their familiarity with the study procedures, laboratory space and the research team. During this visit, baseline HR and mean arterial pressure (MAP) were acquired – through an automated stress test monitor (Tango M2, SunTech Medical, Morrisville, NC, USA) after the participant rested for 15 min in a supine position – and were utilized as the primary measure when distinguishing resting central haemodynamics between groups. The participants’ maximum voluntary contraction (MVC) was acquired, measured using a handgrip force transducer and Biopac software (TSD121C; Biopac, Goleta, CA, USA). While lying supine with their right arm perpendicular to the body, participants were instructed to squeeze the handgrip transducer as hard as possible for 3 s. This process was repeated three times with approximately 1 min of recovery time between attempts. The highest force achieved was considered their MVC and was used to calculate all relative workloads for the study visits. Participants were then familiarized with each procedure of the study protocol to ensure their understanding and comfort with the various tests that would be conducted. Body fat percentage and forearm volume were measured through seven‐site skinfold assessment (Lange Skinfold Caliper, Beata Technology, Inc., Cambridge, MD, USA).

Following the completion of the familiarization visit protocols, each participant was issued an accelerometer (Actigraph GT3X+, Pensacola, FL, USA) to wear for 7 days. They were instructed to complete a 7‐day activity and sleep diary to track these lifestyle behaviours and return the diary at a subsequent study visit.

### 2.4 Study visits

All study visits involved a blood sample and multiple autonomic and vascular function assessments. After a 15‐min supine resting period, capillary blood was collected through a finger stick to examine whole blood superoxide concentration. The participant was then connected to a beat‐to‐beat haemodynamic measurement system (Finometer MIDI, Finapres Medical Systems, Amsterdam, Netherlands) to record systolic blood pressure (SBP), diastolic blood pressure (DBP) and MAP via finger photoplethysmography and HR via electrocardiogram during all assessments. The sensor was attached to the participant's left middle finger and wrist using Velcro straps. HR and MAP were the primary measures for each of the autonomic function assessments, while blood flow and vascular conductance were used to evaluate vascular function.

### 2.5 Oxidative stress assessment

At the start of each study visit, a capillary blood sample was acquired via finger stick. The concentration of the OXS marker, superoxide, was measured using a cyclic hydroxylamine (CMH; Noxygen Science Transfer & Diagnostics, Elzach, Germany) spin probe and electron paramagnetic resonance (EPR; e‐scan, Bruker; Karlsruhe, Germany). To create the spin probe, a solution of 0.1 mM CMH was dissolved in 100% ethanol. This solution was mixed with the blood sample in a 1:3 ratio and incubated for 1 h at room temperature, then transferred to a 50 µL micropipette for analysis (Decker et al., 2023). The EPR was configured with the following settings: field sweep, 100 G; microwave frequency, 9.79 GHz; microwave power, 21.05 mW; modulation amplitude, 2.39 G; conversion time, 5.12 ms; detector time constant, 1.25 ms; and receiver gain, 3.17.

### 2.6 Autonomic function assessments

#### 2.6.1 Exercise pressor test

An isometric handgrip exercise was employed to investigate potential differences in autonomic regulation between groups. This test consists of a continuous isometric muscle contraction that is thought to activate numerous SNS mediators, specifically through the contributions of central command (via perceived effort) and that of the exercise pressor reflex, driven through skeletal muscle mechano‐ and metabo‐receptors (via changes in muscle length and metabolite buildup, respectively), and modulated through the cardiovagal baroreflex (Grotle et al., 2020). Exaggerated exercise pressor reflex has been related to myriad disease states, including hypertension, peripheral artery disease, heart failure and chronic kidney disease (Grotle et al., 2020).

Using a handgrip dynamometer in their right hand while in supine position with the right arm perpendicular to the body, participants performed a 2‐min continuous, isometric muscle contraction at 30% of their MVC. To determine any potential differences due to PTSD, beat‐by‐beat analysis of MAP and HR was obtained via finger plethysmography throughout the test to quantify central haemodynamic responses to the isometric handgrip exercise.

#### 2.6.2 Cardiovagal baroreceptor sensitivity test

To assess baroreflex sensitivity, specifically how HR is inversely altered to maintain blood pressure, the participants performed a 15‐s Valsalva manoeuvre, holding a mouth pressure of 40 mmHg as assessed using a pressure gauge, followed by a brief (∼60 s) recovery period. Beat‐by‐beat changes in HR (R‐R interval) and SBP were monitored using finger plethysmography and electrocardiogram (Finapres MIDI) during this test. The Valsalva manoeuvre resulted in a brief rise (Phase I) and subsequent substantial fall (Phase II) of MAP, and following cessation of the manoeuvre, a brief drop (Phase III) and then substantial rise (Phase IV) in SBP. The slope of SBP and the R‐R intervals during Phases II and IV provides a measure of cardiovagal baroreceptor sensitivity, with lower slopes representing lower sensitivity. The test was repeated three times, and the slopes (*r* ^2^ ≥ 0.7) of each measurement were averaged.

### 2.7 Vascular function assessment

#### 2.7.1 Rapid onset vasodilation assessment

To assess microvascular function, specifically local vasodilatory control of blood flow, the ROV technique was employed. Prior studies using pharmacological blockade and α‐adrenergic stimulation demonstrate that ROV responses in young adults are predominantly mediated by local dilatory mechanisms with minimal neural contribution (Carlson et al., 2008). Sympathetic vasoconstriction can influence this process, though ROV‐induced increases in blood flow at 30% MVC have been reported to attenuate sympathetic‐induced vasoconstriction (phenylephrine), maintaining blood flow responses (Casey & Joyner, 2012). In this study, participants performed a 2‐s handgrip contraction at 30% of their MVC using their right hand, with the right arm perpendicular to the body while lying in a supine position. Ultrasonography (LOGIQ e, GE Healthcare, Chicago, IL, USA) was used to measure blood velocity and brachial artery diameter in the contracting arm over 30 cardiac cycles following the contraction. These measurements were combined with MAP responses to evaluate local vascular conductance.

### 2.8 Statistical analysis

Multiple power analyses based on prior published work examining baroreceptor sensitivity (Phase IV slopes) (Fonkoue, Marvar, et al., 2018), SBP responses to isometric exercise (30% MVC; Berbrier et al., 2024) and vascular conductance responses to single handgrip contractions (30% MVC; Carlson et al., 2008) were conducted to determine the required number of participants needed per group to detect significant differences in each outcome variable. Assuming a two‐sided significance level of α = 0.05, the smallest sample size required to provide adequate power (0.80) to detect similar changes in each outcome variable was 11 participants per group. For the comparison of PTSD and control groups, an independent samples Student's *t*‐test was used for all vascular and autonomic nervous system assessments, including microvascular function, cBRS and exercise pressor test. Pearson correlation analyses were also performed to examine associations between PTSD symptom severity and each outcome variable.

## 3 RESULTS

### 3.1 Demographics and resting physiological measures

This study revealed that both healthy controls (CTRL) and individuals exhibiting symptoms of PTSD were not significantly different in age (*P* = 0.48), height (*P* = 0.58), weight (*P* = 0.51), body mass index (BMI; *P* = 0.51), body fat percentage (*P* = 0.84), and maximum *h* and grip voluntary contraction (*P* = 0.75), underscoring the comparability of physical characteristics across the study population (Table 1). Of the 38 female participants in the study, 19 were currently employing birth control methods during the study period (intrauterine device: CTRL, *n* = 2; PTSD, *n* = 3; oral contraceptives, CTRL, *n* = 3; PTSD, *n* = 2). Differences were identified in resting physiological measures. When compared to CTRL, the PTSD group exhibited higher SBP (*P* < 0.01) and MAP (*P* = 0.04), but not DBP (*P* = 0.13) and HR (*P* = 0.44) (Table 1). Moreover, assessments via mental health questionnaires (Beck Anxiety Index [BAI], Beck Depression Index [BDI], State‐Trait Anxiety Index [STAI], and the PTSD Checklist 5 [PCL‐5]) revealed significant differences between the groups (all *P* < 0.01), further delineating the psychological and emotional contrasts inherent to PTSD (Table 1). Sleep and physical activity measures were revealed to be not significantly different between groups when examined for sleep quality (sleep efficiency: *P* = 0.7; total sleep time: *P* = 0.68), sleep regularity (sleep efficiency standard deviation: *P* = 0.18; total sleep time standard deviation: *P* = 0.06), average daily sedentary time (*P* = 0.52), moderate‐to‐vigorous activity (*P* = 0.53) and step count (*P* = 0.30) (Table 2). PTSD symptom severity was not significantly correlated with any resting central haemodynamic (*r* = 0.12–0.26, *P* = 0.10–0.43) or sleep/physical activity variables (*r* = 0.01–0.26, *P* = 0.10–0.95) when examined across all participants.

**TABLE 2:** Sleep and physical activity data.

	CTRL (*n* = 22)	PTSD (*n* = 22)	*P*
Sleep quality			
Sleep efficiency (%)	86 ± 7	85 ± 8	0.74
Total sleep time (min/night)	417 ± 66	409 ± 65	0.68
Sleep regularity			
SE SD	4 ± 2	5 ± 3	0.18
TST SD	56 ± 24	75 ± 36	0.06
Average daily activity			
Sedentary (min/day)	961 ± 152	923 ± 172	0.52
Total MVPA (min/day)	44 ± 19	40 ± 20	0.53
Step count (steps/day)	6258 ± 2790	7274 ± 3286	0.30

Values are expressed as means ± SD. Statistical analysis was conducted using an independent samples *t*‐test (CTRL vs. PTSD). CTRL, control; MVPA, moderate to vigorous daily physical activity; PTSD, post‐traumatic stress disorder; SE SD, standard deviation of sleep efficiency; TST SD, standard deviation of total sleep time.

### 3.2 Oxidative stress

**FIGURE 1:**
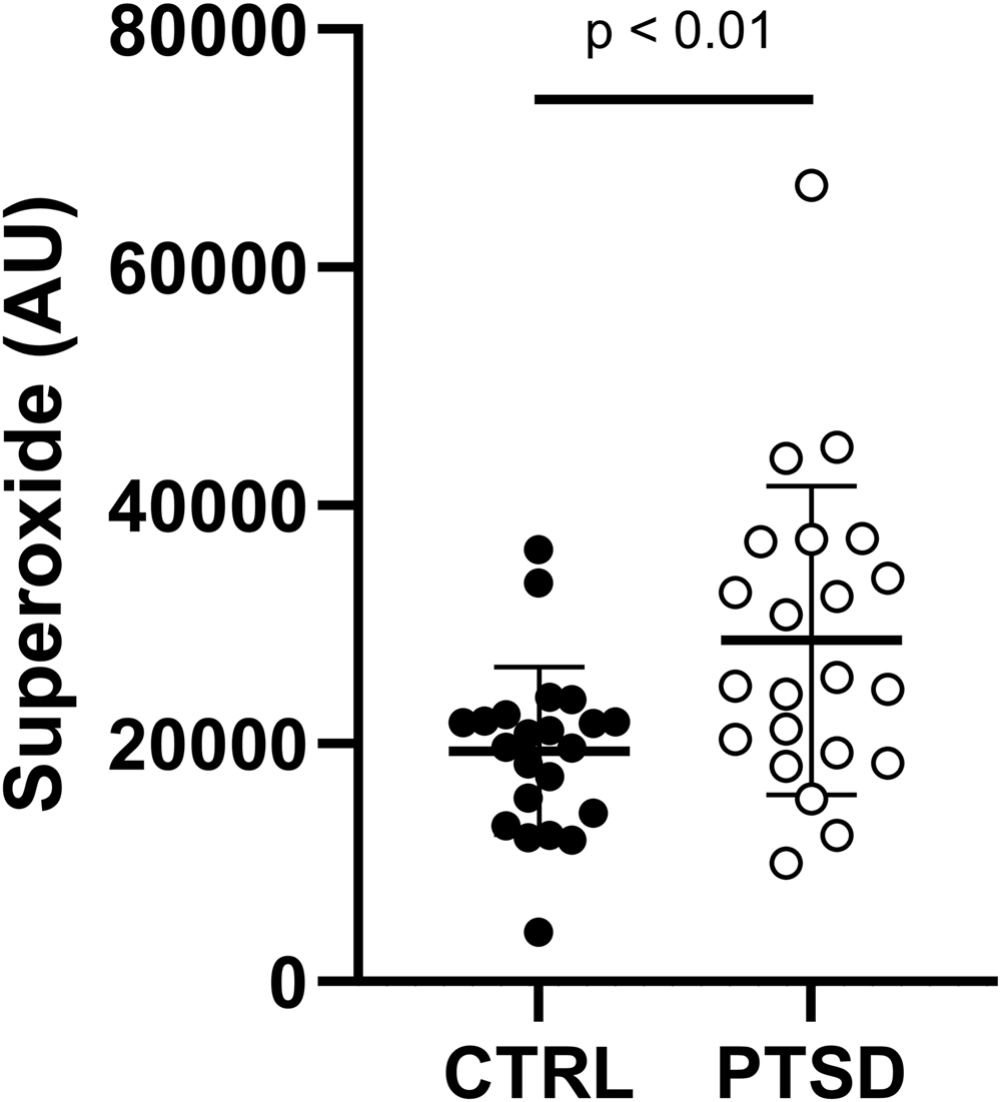
Whole blood superoxide production for participants with (PTSD, *n* = 22; 19 females/3 males) and without (CTRL, *n* = 22; 19 females/3 males) post‐traumatic stress disorder. Data were analysed with an independent samples *t*‐test and are represented as means ± standard deviation as well as individual data points. CTRL, control; PTSD, post‐traumatic stress disorder.

A significant difference in whole blood superoxide levels was revealed between PTSD and CTRL (*P* < 0.01), with higher superoxide levels in the PTSD group (Figure 1). PTSD symptom severity was significantly correlated with whole blood superoxide (*r* = 0.35, *P* = 0.02) when examined across all participants.

### 3.3 Autonomic function: Exercise pressor test

Blood pressure and HR responses evaluated during the final minute of the 2‐min isometric handgrip test done at 30% MVC did not uncover statistically significant differences in the changes observed from baseline to the final minute of isometric handgrip exercise (Table 3). Indeed, the difference in systolic (∆SBP; *P* = 0.66), DBP (∆DBP; *P* = 0.67), MAP (∆MAP; *P* = 0.53), and HR (∆HR; *P* = 0.67) from baseline to the final minute of exertion exhibited no significant differences between groups (Table 3). PTSD symptom severity was not significantly correlated with blood pressure or HR responses to isometric handgrip exercise (*r* = 0.02–0.16, *P* = 0.28–0.86) when examined across all participants.

**TABLE 3:** Autonomic function: exercise pressor test.

	CTRL (*n* = 22)	PTSD (*n* = 22)	*P*
∆SBP	12 ± 9	11 ± 9	0.66
∆DBP	9 ± 6	8 ± 6	0.67
∆MAP	12 ± 7	10 ± 8	0.53
∆HR	5 ± 4	6 ± 8	0.67

Values are expressed as means ± SD. Statistical analysis was conducted using an independent samples *t*‐test. CTRL, control; DBP, diastolic blood pressure; HR, heart rate; MAP, mean arterial pressure; PTSD, post‐traumatic stress disorder; SBP, systolic blood pressure.

### 3.4 Autonomic function: Cardiovagal baroreflex function

**FIGURE 2:**
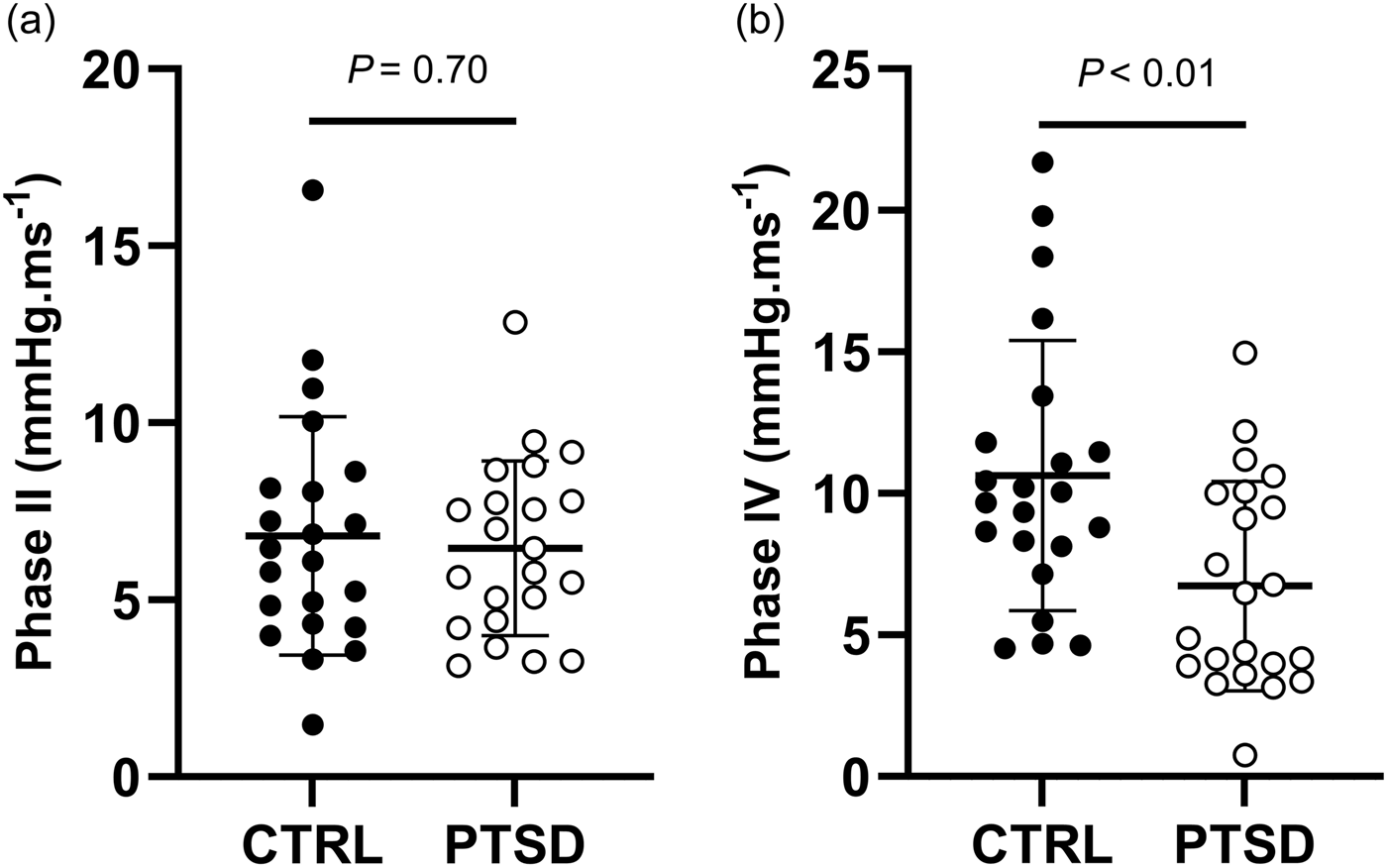
Phase II (a) and Phase IV (b) cardiovagal baroreflex sensitivity for participants with (PTSD, n = 22; 19 females/3 males) and without (CTRL, n = 22; 19 females/3 males) post‐traumatic stress disorder. Data were analysed with an independent samples t‐test and are represented as means ± standard deviation as well as individual data points CTRL, control; PTSD, post‐traumatic stress disorder.

Evaluation of the cBRS revealed no significant differences between the groups in cBRS during Phase II of the reflex (Figure 2). However, a notable divergence emerged during Phase IV. PTSD demonstrated a significantly slower HR response to elevations in SBP when compared to CTRL (*P* < 0.01) (Figure 2). PTSD symptom severity was not significantly correlated with either Phase II or Phase IV cBRS (*r* = 0.02–0.22, *P* = 0.15–0.85) when examined across all participants.

### 3.5 Microvascular function: Rapid onset vasodilation test

Following an ROV induced by 2 s of handgrip contraction at 30% MVC, no significant differences in cardiovascular responses between PTSD and CTRL emerged when examined as vascular conductance area under the curve (VC‐AUC; *P* = 0.16) or peak change in VC (∆VC peak; *P* = 0.30) (Table 4). In addition, no significant differences in BA diameter (*P* = 0.70), BF‐AUC (*P* = 0.07), or peak change in BF (*P* = 0.13) were observed (Table 4). PTSD symptom severity was not significantly correlated with microvascular function (*r* = 0.09–0.15, *P* = 0.34–0.54) when examined across all participants.

**TABLE 4:** Microvascular function: rapid onset vasodilation test.

	CTRL (*n* = 22)	PTSD (*n* = 22)	*P*
Resting BA diameter (mm)	3.2 ± 0.5	3.3 ± 0.6	0.70
BA BF			
Peak change from BL (mL min^−1^)	84 ± 46	116 ± 88	0.13
AUC (mL)	14 ± 13	26 ± 27	0.07
VC			
Peak change from BL (mL min^−1^ mmHg^−1^)	1.1 ± 0.1	1.3 ± 0.8	0.16
AUC (mL mmHg^−1^)	0.2 ± 0.2	0.3 ± 0.2	0.30

Values are expressed as means ± SD. Statistical analysis was conducted using an independent samples *t*‐test. AUC, area under the curve; BA, brachial artery; BF, blood flow; CTRL, control; PTSD, post‐traumatic stress disorder; VC, vascular conductance.

## 4 DISCUSSION

### 4.1 Major findings

This study sought to identify the presence of specific autonomic and/or vascular dysfunction in young adults with PTSD, a cohort less impacted by medication usage and PTSD‐linked negative lifestyle behaviours, with the goal of identifying potential early signs of elevated risk for CVD development. The major findings of this study were that young adults with PTSD displayed elevated OXS and blunted cBRS during the hypertensive phase (Phase IV) of the Valsalva manoeuvre when compared to a non‐PTSD, age‐ and sex‐matched control group. Further, this study revealed that exercise‐induced blood pressure and local vascular responses were intact in young adults with PTSD, highlighting a preservation of this function.

The investigation extends our prior work in young adults with PTSD by directly exploring autonomic versus vascular contributions to early cardiovascular maladaptations (Weggen et al., 2021, 2024). Through direct quantification of baroreflex function via Valsalva manoeuvre in parallel with an assessment of vascular conductance, the present study uniquely distinguishes autonomic dysfunction from local vascular mechanisms. The findings implicate impaired cBRS as a potential early maladaptation in young adults with PTSD, occurring in the absence of overt vascular dysfunction.

### 4.2 Demographics

Despite recruiting equally across sexes, most PTSD volunteers in this study were female, necessitating a predominantly female control group. This outcome was unsurprising, given the 1‐year prevalence of PTSD among civilian women is 6–8% compared to 2–4% for men (Goldstein et al., 2016) and Virginia Commonwealth University, our primary recruitment site, has a student body that is ∼64% female (Virginia Commonwealth University, 2024). With that stated, the PTSD and control groups were well matched for age, physical characteristics, physical activity and sleep, minimizing confounding influences on cardiovascular outcomes. Given sleep disturbance is a hallmark PTSD symptom, differences in objective sleep outcomes between groups were anticipated. However, 7 days of sleep monitoring revealed no significant group differences in latency, efficiency, wake after sleep onset, number or duration of awakenings or regularity. Discrepancies with prior studies, may be attributable, in part, to sleep assessment methodology and/or the moderating influence of age‐related physiological resilience. Shorter, more sensitive measurements may detect transient, state‐dependent disturbances (Habukawa et al., 2007), whereas longer‐duration actigraphy captures a habitual sleep continuity that may remain intact in younger populations (Lewis et al., 2020; Schenker et al., 2023).

As expected, mental health measures differed significantly between groups. These results underscore PTSD's profound psychological burden and its well‐documented association with increased risk for comorbid mental health conditions (Galatzer‐Levy et al., 2013). Despite demographic matching, PTSD participants exhibited higher resting SBP, MAP and HR than controls, reflecting autonomic dysregulation likely driven by sympathetic overactivity and/or parasympathetic withdrawal (Ahmed et al., 2024; Williamson et al., 2015). Prior work shows PTSD with elevated BP is linked to diminished parasympathetic activity and abnormal stress reactivity (Fonkoue, Norrholm, et al., 2018).

### 4.3 Oxidative stress

We directly assessed OXS in young adults with PTSD by measuring whole blood superoxide production and found significantly higher levels in PTSD compared to controls. Importantly, PTSD symptom severity was positively correlated with whole‐blood superoxide production, indicating that OXS increases in parallel with clinical symptom burden. This supports prior reports of elevated ROS in PTSD (Ceprnja et al., 2011; Miller et al., 2018). Unlike prior studies from other groups that relied on indirect markers of OXS (e.g., protein or lipid oxidation), our direct measurement provides unique evidence of OXS in this population. Elevated ROS can promote neuroinflammation and amygdala dysfunction (Lee et al., 2022; Miller et al., 2018), driving excessive sympathetic and hypothalamic–pituitary–adrenal (HPA) axis activity that further amplifies OXS in a maladaptive cycle (Ghaemi Kerahrodi & Michal, 2020). Beyond the brain, OXS may be a major contributor to long‐term cardiovascular health disruption as it is linked to negative alterations of vascular homeostasis by reducing vasodilation and promoting vasoconstriction, contributing to microvascular impairment, endothelial dysfunction and arterial stiffness, all of which are linked to CVD (Wray et al., 2017). Further, OXS has been linked to autonomic dysfunction in the form of elevated sympathetic nerve activity stemming from factors such as muscle afferent sensitization, baroreflex dysfunction, and/or adrenergic receptor sensitization (Koba et al., 2013; Lanfranchi & Somers, 2002; Park et al., 2017). While our prior work demonstrated improvements in vascular function following acute antioxidant supplementation (Weggen et al., 2024), the role of OXS was inferred rather than directly measured, limiting our ability to determine whether elevated OXS is a mechanistic driver. The present findings complement and extend those observations by directly quantifying whole blood superoxide production, providing more definitive evidence of OXS involvement.

### 4.4 Autonomic function: Cardiovagal baroreflex sensitivity

Assessment of cBRS revealed diminished autonomic modulation capacity in the PTSD group, indicated by inadequate HR adjustments to stabilize a rapid rise in blood pressure (Phase IV) when compared to the CTRL group. This impairment compromises maintenance of haemodynamic stability to hypertensive stimuli, which can significantly increase long‐term cardiovascular risks (Hockin et al., 2021). Studies in myriad populations demonstrate that impaired baroreflex sensitivity disrupts autonomic regulation, leading to adverse cardiovascular‐related outcomes (Bruno et al., 2012; Chesterton et al., 2005). Indeed, inadequate adjustments in HR and vascular tone can result in transient and/or prolonged periods of elevated blood pressure (Charkoudian & Rabbitts, 2009), a well‐established risk factor for heart disease (Fuchs & Whelton, 2020; Yano et al., 2018). This continuous strain on the cardiovascular system, stemming from autonomic imbalances, can lead to structural and functional changes in the heart and blood vessels, further increasing cardiovascular risk (Drazner, 2011; Saheera & Krishnamurthy, 2020). Although prior work has demonstrated impaired baroreflex sensitivity in PTSD (Fonkoue et al., 2020), the present study extends these findings to a younger, otherwise healthy cohort and provides additional specificity by identifying a selective impairment during the hypertensive phase (Phase IV) of the Valsalva manoeuvre. These findings represent an incremental but important refinement of existing knowledge by demonstrating that cardiovagal baroreflex impairment is phase‐specific rather than global.

Specific to both OXS and cBRS, prior work by our laboratory highlighted a significant relationship with these variables and sleep irregularity (Richardson et al., 2026). In the current study, although the between‐group difference in total sleep time variability only approached statistical significance, sleep variability remains a plausible contributing factor to the observed autonomic and OXS differences, particularly given prior evidence linking greater sleep variability with reduced cBRS and elevated superoxide concentrations in young adults with chronic anxiety. However, this interpretation should be made cautiously given that not all participants met recommended actigraphy duration criteria (>7 days) for robust sleep regularity assessment.

### 4.5 Autonomic function: Exercise pressor test

Contrary to our hypothesis, we did not demonstrate a significant difference in MAP or HR between PTSD and CTRL during static handgrip exercise. The isometric small muscle mass exercise test, which is designed to activate the exercise pressor response, showed no significant differences between groups in either HR or MAP during the final minute of 2 min of sustained exercise. The exercise pressor reflex is a neural feedback system that works in conjunction with central command to ensure blood flow is redistributed to working tissue and maintain adequate blood pressure during exercise (Kaufman & Hayes, 2002; Mark et al., 1985; Rowell & O'Leary, 1990). This reflex is initially triggered by central command and through mechanical and metabolic stimulation of group III and IV afferents during muscle contraction (Kaufman & Hayes, 2002). Additional adjustments are made by arterial and cardiopulmonary baroreflexes (Fadel & Raven, 2012). In the present study, HR and MAP responses during the final minute of exercise were comparable between groups, so it appears that these mechanisms are not impaired in the PTSD group.

This finding contrasts with our laboratory's prior work demonstrating elevated MAP responses during rhythmic, isotonic handgrip exercise in young adults with PTSD (Weggen et al., 2024). It may be plausible that differences in exercise modality (isometric vs. isotonic) contributed to the observed discrepancies. These modalities may differentially engage central command, the exercise pressor reflex and baroreflex buffering (Grotle et al., 2020), which may alter cardiovascular responses in this population. Accordingly, the present findings should be interpreted with caution, and future studies are needed to systematically evaluate modality‐specific and intensity‐dependent influences on autonomic and haemodynamic regulation in this population.

This finding contrasts with our laboratory's prior work that highlighted elevated MAP responses during rhythmic, isotonic handgrip exercise in young adults with PTSD (Weggen et al., 2024). Indeed, it was hypothesized that elevated sympathetic nerve activity would be present and occur secondary to PTSD‐associated factors such as muscle afferent sensitization, baroreflex dysfunction, and adrenergic receptor sensitization (D'Souza et al., 2024; Fonkoue et al., 2020; Park et al., 2017). Although the present study focused primarily on autonomic reflex function rather than perfusion‐normalized conductance during sustained exercise, a greater exercise‐induced MAP response was still hypothesized. This discrepancy may be explained, at least in part, by differences in exercise modality and intensity, as the prior rhythmic, isotonic handgrip protocol was completed at a greater intensity relative to maximum workload (∼65%). This greater intensity has elicited greater metabolic demand and overall cardiovascular strain compared to the sustained isometric handgrip task completed at 30% MVC and used in the present study, thereby provoking a more robust MAP response in participants with PTSD. A more thorough evaluation of intensity‐dependent alterations in MAP responses to both isotonic and isometric exercise in this population is needed.

### 4.6 Vascular function

The results from the vascular function assessment provide insight into how the vasculature responds to a 2‐s forearm contraction at 30% of MVC. Blood flow and vascular conductance responses were not significantly different between PTSD and control groups, indicating that acute cardiovascular responses to this brief physical stressor were not different across conditions. These findings suggest that, at least under the transient workload assessed by the ROV test, vascular function appears preserved in young adults with PTSD. In light of our laboratory's earlier findings of macro‐ and microvascular function in response to ischaemia and handgrip exercise (Weggen et al., 2021, 2024), this outcome may highlight the predominant role of autonomic dysfunction in these lower responses. Indeed, our laboratory previously reported reduced hyperaemic responses (evidence of microvascular dysfunction) to ischaemia and passive leg movement, which have both been shown to be negatively influenced by elevations in resting sympathetic nerve activity (Atkinson et al., 2015; Hanson et al., 2025; Venturelli et al., 2022). In contrast, the ROV technique has been shown to be sympatholytic (Crecelius et al., 2013), which, based on its ability to overcome elevations in resting sympathetic nerve activity, provides strong evidence for preserved local vascular control in this population. Further, prior work by our laboratory reported lower vascular conductance during handgrip exercise in young adults with PTSD (Weggen et al., 2024). This finding was driven by elevations in MAP responses, which may reflect low perfusion (vascular dysfunction) (Daley et al., 2003) and/or elevated sympathetic nerve activity secondary to PTSD‐associated factors such as muscle afferent sensitization, baroreflex dysfunction and adrenergic receptor sensitization (D'Souza et al., 2024; Fonkoue et al., 2020; Park et al., 2017). Indeed, the preservation of ROV in the present study suggests intact local, contraction‐induced vasodilatory mechanisms, whereas previously observed reductions in vascular conductance during sustained handgrip exercise may instead reflect altered centrally mediated sympathetic vasoconstrictor modulation during prolonged stress rather than impairment in intrinsic microvascular dilatory capacity (Weggen et al., 2024). Thus, these results suggest that local vascular control remains intact in young adults with PTSD, but could also could represent direct discrepancies relative to our prior work (Weggen et al., 2024). As the present findings derive from a PTSD cohort independent of our prior work, this may further emphasize the need for continued investigation and replication to clarify the consistency and context‐dependence of autonomic and vascular responses in young adults with PTSD.

### 4.7 Limitations

The participant pool was composed primarily of young, premenopausal women with PTSD, which limits the generalizability of our findings. These results may not extend to men, perimenopausal or postmenopausal women, or young women with other mental health disorders. Although study visits were scheduled during the early follicular phase of the menstrual cycle, individual variations in hormone levels, particularly oestrogen, which plays an important role in antioxidant defence, were not confirmed by blood testing and may have influenced outcomes. As PTSD is often comorbid with anxiety and depression it should be noted that elevated depressive and anxiety symptom burden may independently contribute to autonomic dysregulation and OXS, potentially influencing the present findings either alone or synergistically with PTSD symptomatology. Although this study was not powered to disentangle the independent contributions of each psychological construct, future investigations using multivariate modelling approaches will be important to clarify their distinct versus overlapping physiological effects. Finally, while comorbid medical conditions were minimized, a small number of PTSD participants (*n* = 3) were taking prescription medications, which could have introduced additional variability. Excluding the three participants taking prescribed medications did not alter the primary findings of elevated OXS and lower cBRS, which remained statistically significant.

### 4.8 Conclusion

The overarching goal of this study was to examine key cardiovascular and autonomic markers linked to future development of CVD. This investigation aimed to provide insight into the mechanisms driving the increased CVD risk common among young individuals with PTSD to inform potential early interventions that could mitigate these effects. Our results indicate that although BP regulation and vascular function responses to acute exercise remain unaltered, young adults with PTSD exhibit elevated OXS and baroreflex dysfunction in responses to a hypertensive stimulus, potentially reflecting an early physiological maladaptation to PTSD in this young cohort.

## AUTHOR CONTRIBUTIONS

Jennifer B. Weggen and Ryan S. Garten drafted the manuscript. All authors performed data collection. Jennifer B. Weggen and Ryan S. Garten conducted formal analysis. All authors revised the manuscript. All authors have read and approved the final version of this manuscript and agree to be accountable for all aspects of the work in ensuring that questions related to the accuracy or integrity of any part of the work are appropriately investigated and resolved. All persons designated as authors qualify for authorship, and all those who qualify for authorship are listed.

## CONFLICT OF INTEREST

None declared.

## FUNDING INFORMATION

None.

## Data Availability

The data that support the findings of this study are available from the corresponding author upon reasonable request.
